# Identification of novel splice mutation in *SMAD3* in two Cypriot families with nonsyndromic thoracic aortic aneurysm. Two case reports

**DOI:** 10.1002/mgg3.1378

**Published:** 2020-06-29

**Authors:** Anna Keravnou, Evy Bashiardes, Vassilis Barberis, Kyriaki Michailidou, Marinos Soteriou, George A. Tanteles, Marios A. Cariolou

**Affiliations:** ^1^ Department of Cardiovascular Genetics and The Laboratory of Forensic Genetics The Cyprus Institute of Neurology and Genetics Nicosia Cyprus; ^2^ Cyprus School of Molecular Medicine The Cyprus Institute of Neurology and Genetics Nicosia Cyprus; ^3^ Department of Cardiology and Cardiovascular Surgery American Medical Center Nicosia Cyprus; ^4^ Biostatistics Unit The Cyprus Institute of Neurology & Genetics Nicosia Cyprus; ^5^ Clinical Genetics Clinic The Cyprus Institute of Neurology & Genetics Nicosia Cyprus

**Keywords:** Cyprus, nonsyndromic familial thoracic aortic aneurysm and dissection, *SMAD3*, targeted next‐generation sequencing

## Abstract

**Background:**

Thoracic aortic aneurysm and dissection (TAA/D) represents a potentially lethal disease group characterized by an increased risk of dissection or rupture. Only a small percentage (approximately 30%) of individuals with nonsyndromic familial TAA/D have a pathogenic variant in one of the genes that have been found to be associated with the disease.

**Methods:**

A targeted sequencing panel and direct sequencing approach were used to identify causative mutations in the index patients and other family members.

**Results:**

In this study we report two apparently unrelated Cypriot families with nonsyndromic familial TAA/D. The proband A is a female patient diagnosed with TAA/D and intracranial aneurysm and opted for an elective intervention. The proband B is a male patient who was diagnosed with TAA/D and underwent cardiac surgery. Sequencing analysis identified a novel splice site variant (c.871+1G>A) in *SMAD3* which is shown to be associated with the disease. Analysis of mRNA from the patient's tissue confirmed aberrant splicing and exon 6 skipping.

**Conclusion:**

Our findings expand the mutation spectrum of variants that have been shown to be associated with nonsyndromic familial TAA/D. This study demonstrates the importance of a comprehensive clinical and genetic evaluation aiming at early diagnosis and intervention.

## Graphical abstract

In this study we report two apparently unrelated Cypriot families with nonsyndromic familial thoracic aortic aneurysm and dissection associated with a novel variant in SMAD3. Analysis of mRNA from patients’ tissue confirmed aberrant splicing and exon 6 skipping. This study demonstrates the importance of a comprehensive clinical and genetic evaluation aimed at early diagnosis and intervention.

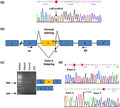

## 1 INTRODUCTION

Thoracic aortic aneurysm and dissection (TAA/D) is a potentially lethal disease since a ruptured aneurysm can lead to severe internal bleeding (Landenhed et al., 2015). Aortic dissections are classified into two main types: type A and type B involving the ascending and descending part of the aorta, respectively. Type A is more common than type B and it is usually associated with more severe complications that may lead to sudden death due to the tendency to cause coronary artery occlusion, spontaneous rupture into the pericardium resulting in cardiac tamponade, or dissection into the aortic valve which leads to aortic insufficiency. Type B dissections are characterized by a lower incidence of spontaneous rupture and are therefore less commonly associated with medical emergency (Lempel et al., 2014). The prevalence of TAA/Ds in the general population is estimated to be approximately 1% (Verstraeten, Luyckx, & Loeys, 2017). TAA/D is mostly inherited as an autosomal dominant trait which is mainly caused by mutations in genes encoding proteins involved in the transforming growth factor‐β signaling pathway [*SMAD3* (OMIM 603109), *TGFB2* (OMIM 090220), *TGFBR1* (OMIM 190181) and *TGFBR2* (OMIM 190182); the smooth muscle cell contractile apparatus (*ACTA2* (OMIM 611788), *MYH11* (OMIM 160745), *MYLK* (OMIM 613780) and *PRKG1* (OMIM 615436)] and the extracellular matrix (*FBN1*) (OMIM 154700) (Brownstein et al., 2017; Jondeau & Boileau, 2014; Keravnou et al., 2018). In the last two decades, 30 genes have been identified that contribute to the development of TAA/D and approximately 30% of patients with nonsyndromic familial TAA/D have a pathogenic mutation in one or more of these genes (Brownstein et al., 2018). However, the genetic cause for a large number of cases remains unknown. Therefore, further studies are needed to identify novel genes and variants associated with TAA/D. The early identification of mutations and variants associated with TAA/D is critical for the asymptomatic individuals in order to prevent sudden death (Milewicz & Regalado, 2003).

In this study we report two apparently unrelated Cypriot families with nonsyndromic familial TAA/D associated with a novel variant in *SMAD3*. When preparing this case report the CARE Guidelines: Consensus‐based Clinical Case Reporting Guideline Development were taken into account (Gagnier et al., 2013).

## 2 MATERIALS AND METHODS

### 2.1 Ethics statement

The study has been approved by the Cyprus National Bioethics Committee and all the study participants gave informed, written consent.

### 2.2 DNA extraction and analysis

DNA samples were extracted from peripheral blood samples using the QIAamp Blood Midi Kit (Qiagen) according to the manufacturer's instructions. The TruSight Cardio Sequencing panel (Illumina), which includes the most predominant genes with known association to familial aortic aneurysm, was initially used for the identification of possible variants in proband A. Paired‐end sequencing was performed on an Illumina Miseq platform.

### 2.3 Bioinformatic analysis

Sequencing reads were mapped to the reference human genome (GRCh37/hg19) with Burrows Wheeler Aligner (BWA) (Li & Durbin, 2009). Local realignment and base recalibration were performed using the GATK suite software and according to best practices (Auwera et al., 2013). SAMtools software (Li, 2011) was used to retrieve the read depth per‐base. Variants were called using the Haplotype Caller from the GATK (https://software.broadinstitute.org/gatk/). Annotation of variants, Small Nucleotide Variants (SNVs) as well as Insertions and Deletions (InDels) was performed using the ANNOVAR software. Five computational tools have been employed for the task of pathogenicity prediction, including PolyPhen2, SIFT, MutationTaster, PROVEAN and VEP. To assess the effect of the identified splice site variant, three prediction tools (Human Splicing Finder [http://www.umd.be/HSF3/] [Desmet et al., 2009], NNsplice [http://www.fruitfly.org/seq_tools/splice.html] [Reese, Eeckman, Kulp, & Haussler, 1997] and NetGene2 [http://www.cbs.dtu.dk/services/NetGene2/] [Hebsgaard et al., 1996]) were used.

### 2.4 Prioritization of variants detected by targeted next‐generation sequencing

Due to the limited availability of samples from other family members the procedure followed in our laboratory is that initially, all samples are screened by Sanger sequencing for the potential disease‐causing variants that have been previously identified in Cypriot TAA/D patients. If this yields negative results, then the samples are further processed with next‐generation sequencing (NGS). Targeted panel sequencing was applied to the Proband A and the analysis was focused on variants identified in exons (missense, nonsense, frameshift and splice site variants) of the genes (*ACTA2, COL3A1, COL5A1, COL5A2, FBN1, FBN2, MYH11, MYLK, NOTCH1, SLC2A10, SMAD3, SMAD4, TGFB2, TGFB3, TGFBR1, TGFBR2*) that have been previously reported to be associated with TAA/D, in rare variants with <1% frequency in the European population based on the Genome Aggregation Database (gnomAD) (Karczewski et al., 2019), taking into account that gene mutation rates differ greatly in different populations, if not identified in public databases and having consistent scores across the different prediction tools (at least in five).

### 2.5 RNA extraction and RT‐PCR

Total RNA was extracted from homogenized aortic tissue using the RNeasy Fibrous Tissue Mini kit (Qiagen) according to the instructions of the manufacturer. The first‐strand cDNA was synthesized with the ProtoScript First Strand cDNA Synthesis kit (NEB) according to the manufacturer's instructions. Reverse Transcription (RT)‐PCR amplification was carried out by amplification of the cDNA covering exons 5–8 of the SMAD3 mRNA. PCR reactions were performed at 53°C annealing temperature using AmpliTaq Gold^®^ DNA polymerase (Applied Biosystems) and the SMAD3‐Forward: 5′ CTCCAAACCTATCCCCGAAT 3′ and the SMAD3‐Reverse: 5′ AGCGAACTCCTGGTTGTTGA 3′ primers. PCR products were cleaned using QIAquick PCR Purification Kit (Qiagen) according to the manufacturer's instructions.

### 2.6 Sanger sequencing

*SMAD3* was amplified by PCR at 52°C annealing temperature using HotStarTaq DNA Polymerase (Qiagen) and the SMAD3‐Forward: 5′ ACACCCAATGACCCAGTAG 3′ and the SMAD3‐Reverse: 5′ AGAGCACAGCTAAGGATGG 3′ primers. PCR products were cleaned using QIAquick PCR Purification Kit (Qiagen) and followed by cycle sequencing according to the manufacturer's instructions [BigDye Terminator v1.1 Cycle Sequencing Kit (Applied Biosystems)]. The sequencing PCR products were purified using the Performa^®^ DTR Gel Filtration Cartridges (EdgeBio) and then loaded on a 3130xL Genetic Analyzer (Applied Biosystems) with the results analyzed using the Sequencing Analysis 5.2 Software (Applied Biosystems).

### 2.7 In silico protein modeling

The *SMAD3* (wild‐type [WT]) sequence was retrieved from the Ensembl (http://grch37.ensembl.org/) Web site and both WT and mutant, exon 6 deletion, (MUT) submitted to Iterative Threading ASSEmbly Refinement (I‐TASSER) modeling (Yang & Zhang, 2015; Zhang, Freddolino, & Zhang, 2017) to predict full‐length protein structure. I‐TASSER (https://zhanglab.ccmb.med.umich.edu/I‐TASSER/), a structure assembly simulation, was used to predict the secondary structure of the WT and MUT SMAD3. I‐TASSER shows the protein structure based on the closest similarity (with template 1khxA) from the Protein Data Bank (PDB).

## 3 RESULTS

### 3.1 Clinical history

**Figure 1:**
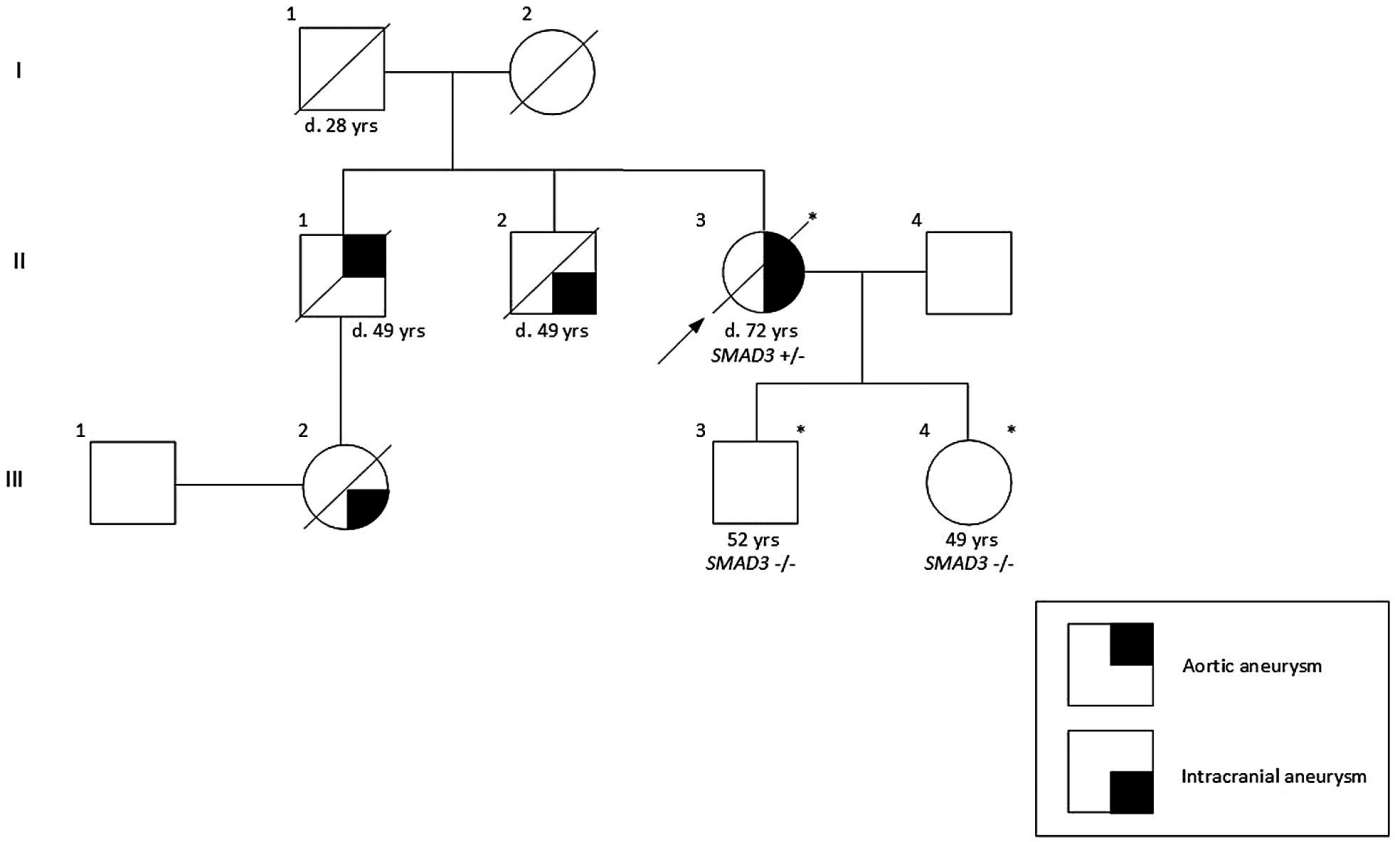
Pedigree of Proband A (II‐3) with c.871+1G>A variant in *SMAD3* (NM_005902.4). Clear symbols represent normal and filled affected individuals. The presence or the absence of the family variants in *SMAD3* is indicated by “+” or “−”. An asterisk represents individuals that were available for this study. Deceased individuals have a slash through the symbol representing them. The index patient is indicated by an arrow. d. = age of death

Pedigree 1 (Figure 1): Proband A (II‐3) with a strong family history of TAAs and intracranial aneurysms (ICAs) was a 69‐year‐old female patient, with a known aneurysm of the aortic root and ascending aorta, that appeared to be expanding over a period of three consecutive years (2013: 44 mm, 2014: 47 mm, 2015: 51 mm) based on serial CT thoracic aorta measurements. Moreover, the patient exhibited symptoms of heart failure (NYHA III) class on the grounds of severe aortic valve regurgitation. Previous medical history included spinal surgery, varicose vein surgery, hysterectomy, cholecystectomy, hyperlipidemia and hypothyroidism. In addition, she was diagnosed with an ICA and opted for an elective intervention. Due to her clinical status, the ICA intervention was postponed and corrective surgery for the aortic aneurysm and valvular disease was initially undertaken. Preoperative coronary angiography showed absence of coronary artery disease. Bio‐Bentall procedure (aortic valve replacement with a bioprosthesis, aortic root, ascending aorta and hemi‐arch replacement with a straight tube graft, and reimplantation of the coronary ostia) was performed at the age of 69 years. Intraoperative findings were a trileaflet aortic valve with intact leaflets and annular dilatation, an aortic root aneurysm with thin walls and an ascending aortic aneurysm of diameters 51 mm and 47 mm respectively, whereas the aortic arch had normal diameter. The patient had a slow but stable recovery, and several months later she underwent coil embolization for her ICA. Unfortunately, the patient then suffered an aortic dissection extending from the aortic arch down to the iliac arteries, and as a result she underwent urgent redo‐sternotomy, aortic arch graft replacement with aortic arch debranching and reconstruction with a trifurcated graft, as well as proximal descending aorta replacement with the Elephant Trunk technique. Intraoperative findings showed an aortic dissection from the mid aortic arch down to the right iliac artery, with the aortic arch measuring 44 mm diameter and the proximal descending aorta 38 mm diameter.

Within this family, sudden death (not investigated) was reported in the proband's father (I‐1) at the age of 28. One of her brothers (II‐1) had died from aortic rupture and another brother (II‐2) from ICA rupture at the age of 49. In addition, a niece (III‐2) had also died from ICA rupture. Subsequently, her two children [(III‐3 (aged 52) and III‐4 (aged 49)] were screened for TAA with echocardiography. Neither of the two children presented with any cardiovascular abnormalities at the time of evaluation.

**Figure 2:**
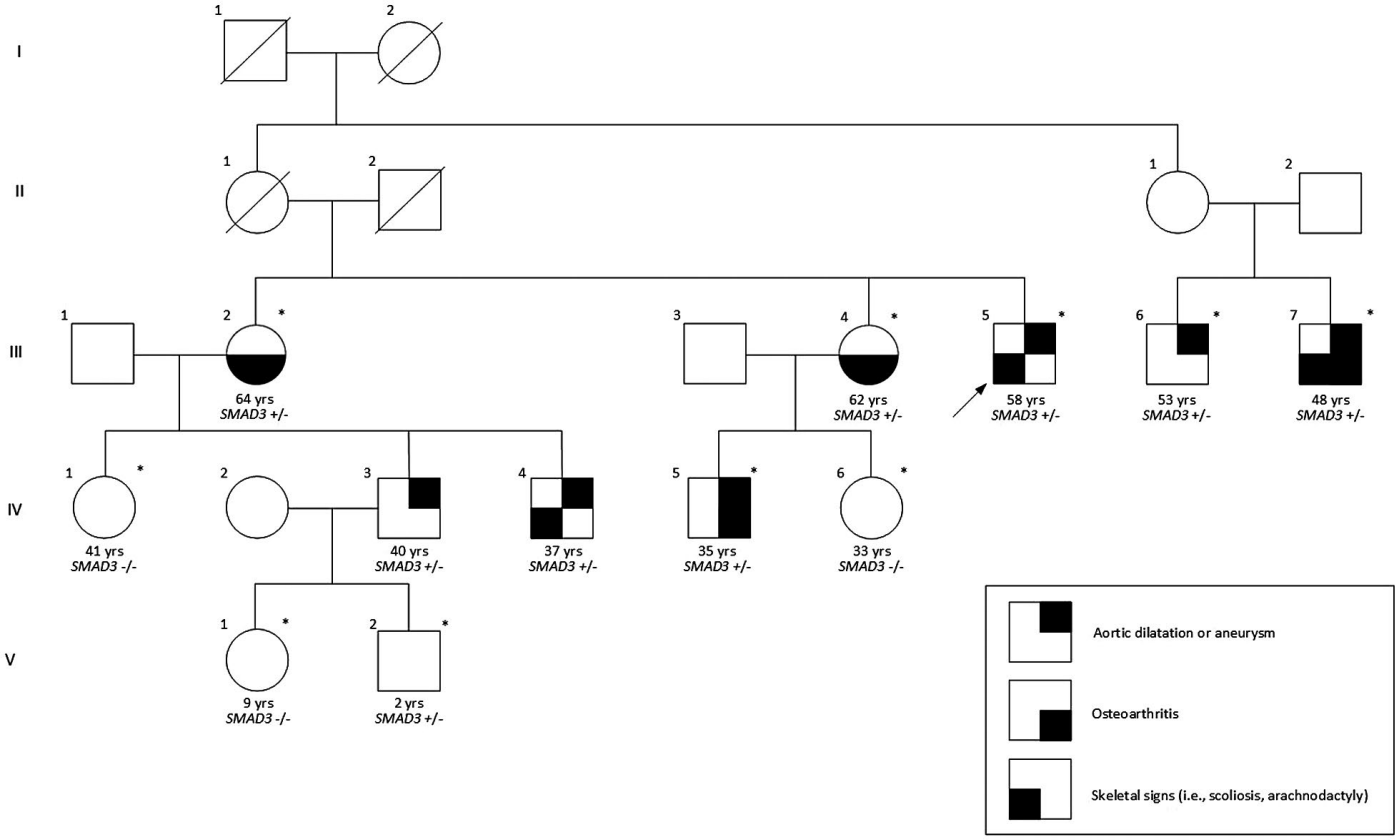
Pedigree of Proband B (III‐5) with eight individuals (Subject III‐2, III‐4, III‐6, III‐7, IV‐3, IV‐4, IV‐5 and V‐2) found to be heterozygous for the c.871+1G>A variant in *SMAD3* (NM_005902.4). The presence or the absence of the family variants in *SMAD3* is indicated by “+” or “−”. The legend indicates the clinical features of the patients. The index patient is indicated by an arrow. An asterisk represents individuals who were available for this study. Deceased individuals have a slash through the symbol representing them

Pedigree 2 (Figure 2): The proband B (III‐5), a male patient who presented with paroxysmal atrial fibrillation (PAF) at the age of 46 years was found following echocardiography and subsequently thoracic aortic CTA to have a large previously unidentified aortic root and ascending aortic aneurysm with a maximum diameter of 60 mm and concomitant moderate aortic valve regurgitation. The patient underwent cardiac surgery that included Bentall procedure (aortic valve, aortic root and ascending aorta replacement with a composite graft) as well as a Maze procedure for the treatment of the PAF. Regarding other clinical findings, this man (III‐5) has a Marfanoid phenotype (tall stature, long upper limbs with ratio of arm span/height >1.05, scoliosis and visual disturbances) but did not formally fulfil criteria for the diagnosis of the syndrome. He also exhibited vitiligo, and he reported that his PAF episodes dated back from his thirties.

The proband B has two nephews (IV‐3 and IV‐4) who are siblings and share similar phenotypic characteristics with proband B. Both were screened for thoracic aortic aneurysm with echocardiography after the diagnosis followed by surgery on their uncle. The elder brother (IV‐3) was examined for the first time in 2009 at the age of 29 and was found to have a moderate‐sized aortic root aneurysm with a diameter of 46 mm. This individual has been followed‐up regularly thereafter by means of echocardiography and thoracic aortic CTA. The aneurysm gradually increased in diameter and reached 53 mm in 2016. The patient underwent an elective aortic root and aortic aneurysm repair with a valve sparing procedure. He was evaluated by a clinical geneticist. He had no history of lens dislocation, dental anomalies or scoliosis. He scored 1 point on the skeletal features’ checklist of the modified Ghent criteria for the diagnosis of Marfan syndrome. His Beighton score was 2/9 and he had negative Walker‐Murdoch and Steinberg signs bilaterally therefore no frank arachnodactyly. His palate was normal as was his uvula. He had mild skin hyperextensibility. At the time of the investigation, neither of the two children of the patient, a nine‐year old‐daughter (V‐1) and a two‐year‐old son (V‐2) presented any clinical manifestations. However, due to the family history, these children were referred for genetic screening. The younger brother (IV‐4) of the proband had a similar phenotype with additional spinal problems, including pectus carinatum and also did not report a history of lens dislocation or dental abnormalities. He too, was examined for the first time at the age of 26 and was found to have a mild aortic root and proximal ascending aorta dilatation with a diameter of 42 mm. He has also been regularly followed‐up thereafter, and over the last 10 years the aneurysm has exhibited slow increase in diameter, with the latest echocardiographic and CT studies showing a diameter of 45‐46 mm. It has been agreed uniformly to continue annual evaluations and to proceed with an elective aortic repair when a threshold of 50 mm is reached. The clinical genetic evaluation indicated a long face with a bone protrusion over the right side of the forehead. His palate was normal as was his uvula. He did not have arachnodactyly. He had an abnormal sternum with pectus excavatum superiorly and a pectus carinatum posteriorly. His ears were soft and had mild skin hyperextensibility. His Beighton score was 0/9 with negative Gorlin sign. Recently, the two brothers were screened for *SMAD3*. The 41‐year‐old sister (IV‐1) of the two siblings did not present neither relevant phenotypic characteristics nor aortic dilatation on echocardiography.

The two sisters (III‐2 and III‐4) of proband B were also clinically examined. Patient III‐2 exhibited spinal abnormalities leading to spinal surgery, paroxysmal arrhythmias, osteoarthritis (diagnosed at the age of 35), diplopia and hernia that had been repaired. Echocardiography revealed normal aortic root and ascending aortic diameters at 35 mm. Similarly, her 62‐year‐old sister (III‐4), despite exhibiting phenotypic characteristics including bifid uvula, arthritis, and spinal abnormalities, did not have on echocardiography aortic dilatation (maximum aortic root diameter 33 mm and ascending aorta 37 mm). The two children of this sister (III‐4) were also examined. The 35‐year‐old son (IV‐5), had bifid uvula, joint hypermobility, arthritis and mitral valve prolapse. He was found to have a mild aortic root dilatation of 38–39 mm on echocardiography. His 33‐year‐old sister (IV‐6) was free of any abnormal clinical or echocardiographic findings.

Finally, two of the cousins of proband B (III‐5) were examined. The first (III‐6), was a 53‐year‐old male who was diagnosed with severe symptomatic mitral regurgitation and underwent mitral valve repair, and also had an aortic root dilatation of 41 mm diameter. The second cousin (III‐7), who had similar phenotypic characteristics to proband B (including arthritis and joint hypermobility), was found to have mild aortic root dilatation of 39 mm. Ascending aorta dimensions were normal in both patients.

### 3.2 Targeted sequencing and genetic analysis

After filtering, targeted NGS results identified one splicing mutation c.871+1G>A (chr15:67473792 G>A) in *SMAD3* (Genbank reference sequences: NM_005902.4 [mRNA] and NP_005893 [protein], Accession and version of reference sequence: NC_000015.9). This variant was not annotated in the major databases, such as the dbSNP (http://www.ncbi.nlm.nih.gov/SNP/), 1000 genome dataset (http://browser.1000genomes.org/), gnomAD (http://gnomad.broadinstitute.org) and Human Genetic Variation Browser databases (http://www.genome.med.kyoto‐u.ac.jp/SnpDB/). The results describing the c.871+1G>A variant were consistent with all five prediction tools (PolyPhen2: Probably Damaging, SIFT: Deleterious, Mutation Taster: Disease causing, PROEVEAN: Deleterious, VEP: Modifier) as having a detrimental effect, suggesting that the site has a key role in the function of *SMAD3*. All three splice site prediction programs (Human Splicing Finder, NNsplice, NetGene2) showed that this sequence alteration abolishes the canonical splice donor site and therefore is likely to disturb normal splicing. This variant was submitted to ClinVar (https://submit.ncbi.nlm.nih.gov/subs/variation_clinvar/SUB6092027/) with ClinVar accession code SCV000992602.

### 3.3 Sanger sequencing analysis

**Figure 3:**
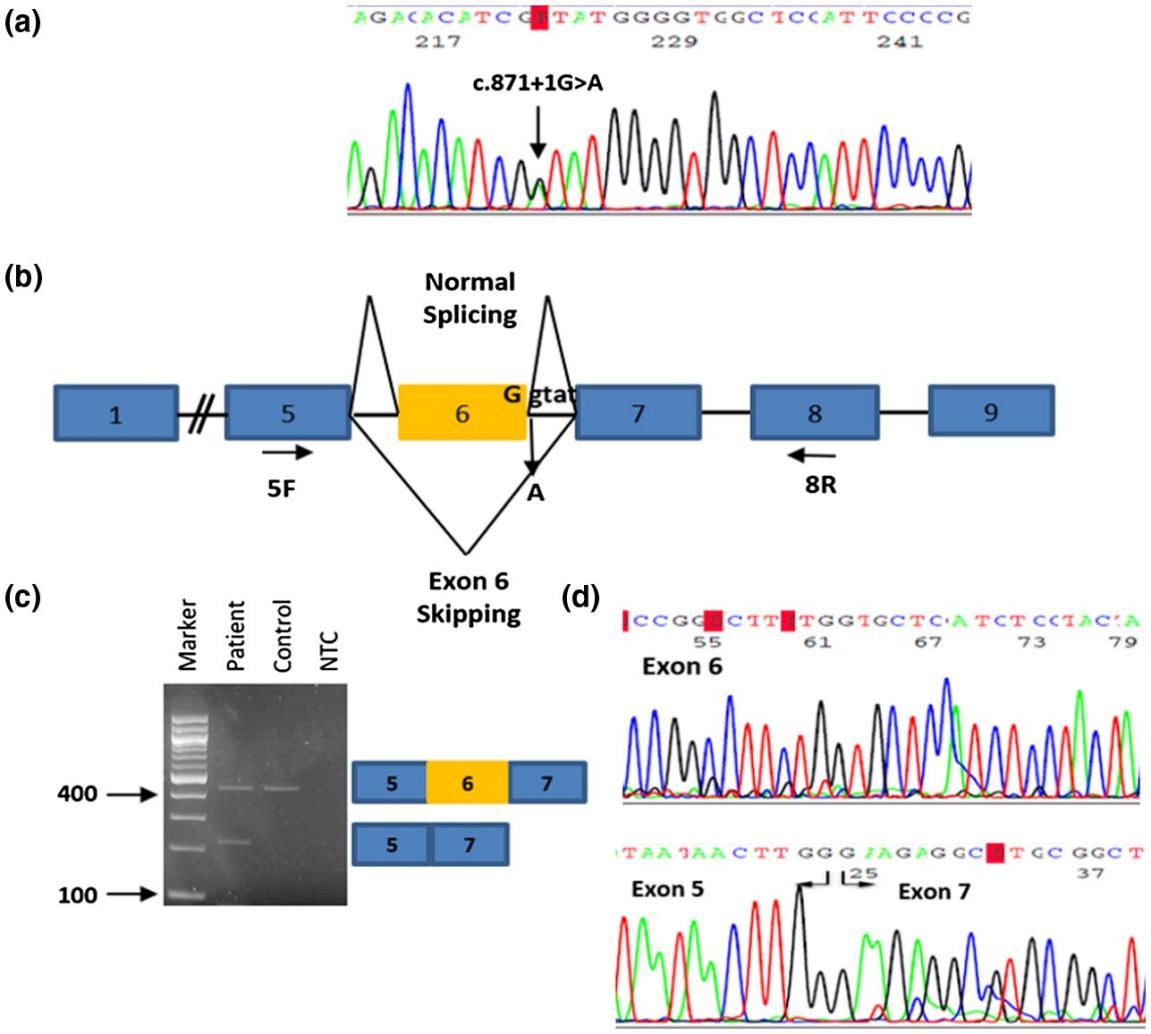
(a) Sanger sequencing electropherogram showing the novel splice site variant (c.871+1G>A) identified in exon 6 of SMAD3 (NM_005902.4) from the gDNA of the patient. (b) Schematic diagram of SMAD3 normal splicing and skipping of exon 6 caused by a variant (c.871+1G>A) in the splice donor site of intron 6. Positions of the primers used for RT‐PCR analysis are indicated by arrows. Exons 1, 5, 7, 8, 9 are represented as blue and exon 6 is represented as yellow. (c) Agarose gel electrophoresis of cDNA amplified from the Proband A (c.871+1G>A) and cDNA from a normal individual (control). The patient's cDNA revealed two bands; the normal (upper) that corresponds to a 437 bp fragment and the mutant (lower) that corresponds to a 224bp fragment size indicating expression from two alleles. The mutation causes exon 6 skipping. NTC = Non‐Template Control. (d) Direct DNA sequence analysis of RT‐PCR fragments (5F–8R) from the affected individual indicates exon 6 skipping

The identified variant was confirmed by Sanger sequencing in proband A (II‐3) (Figure 3a) and was not present in the two unaffected children (III‐3 and III‐4) when screened. Proband B (III‐5) and samples from available family members were also screened by Sanger sequencing for the c.871+1G>A variant in *SMAD3* and results showed that proband B (III‐5), his two sisters (III‐2 and III‐4), the two cousins (III‐6 and III‐7), the one nephew (IV‐5) and the one child (V‐2) of his nephew were also heterozygous of the specific variant whereas the two nieces (IV‐1 and IV‐6) and the other child (V‐1) of his nephew did not show the presence of c.871+1G>A variant in *SMAD3*.

### 3.4 mRNA analysis for the SMAD3 variant

The transcriptional consequence of the splice site mutation (c.871+1G>A) was confirmed by analysis of the mRNA extracted from the aortic tissue of Proband A (Figure 3b). RT‐PCR was performed and cDNA was amplified from exons 5 to 8. Skipping of exon 6, resulting in 213 nucleotides deletion was confirmed by the two bands (224 bp vs. 437 bp for the WT) that appeared on the agarose gel (Figure 3c) and by the cDNA sequencing results. The two DNA fragments were isolated using Montage DNA Gel Extraction Kit (Millipore), according to manufacturer's instructions. Sequence analysis of both strands confirmed that the shorter fragment was lacking the entire exon 6 (Figure 3d) while the larger fragment included exon 6, indicating the normally spliced transcript.

### 3.5 Classification of SMAD3 variant

The pathogenicity of the variant (chr15:67473792 G>A) of *SMAD3* was evaluated using the American College of Medical Genetics and Genomics (ACMG) guidelines (Richards et al., 2015) based on the following criteria: The variant is considered deleterious due to a substitution in a consensus splice site (PVS1). It is novel and absent from a large general population (PM2). Ten individuals, from two different families including a three‐generation family, confirmed cosegregation in a gene with known association to TAA (PP1). Multiple in silico‐based computation analysis supported a deleterious effect on the gene (PP3). The aberrant splicing was also confirmed by RT‐PCR and Sanger sequencing of the product (PS3). Combining the PVS1 criterion with one Strong (PS3), one Moderate (PM2) and one Supporting (PP1), classifies the above *SMAD3* variant as pathogenic.

### 3.6 Predicted secondary structure of SMAD3

**Figure 4:**
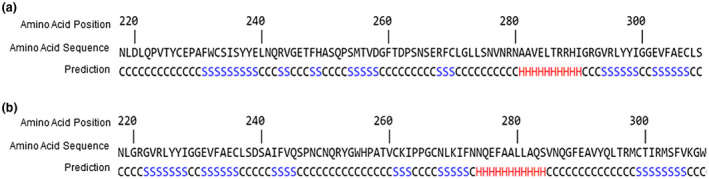
The predicted secondary structure of (a) WT and (b) MUT SMAD3 protein indicating, the corresponding amino acid (220–291) sequence of exon 6 which is absent from mutant SMAD3 using I‐TASSER modeling. C, Coil; H, Helix; I‐TASSER, Iterative Threading ASSEmbly Refinement; S, Strand; WT, wild‐type

The splicing mutation in c.871+1G>A of the SMAD3 is associated with the deletion of the entire exon 6 which most probably leads to defects in protein folding. I‐TASSER modeling prediction tool was used to predict the secondary structure of MUT SMAD3, indicating conformational changes of the protein. The results showed differences in the structure motif between WT (Figure 4a) and MUT (Figure 4b) SMAD3 protein due to the 71 amino acid (220–291) deletions.

## 4 DISCUSSION

The phenotypic spectrum of patients with thoracic aortic aneurysm is characterized by significant intra‐ and inter‐familial variability and varies among carriers of mutations in genes associated to TAA (Hannuksela et al., 2016; Milewicz, Regalado, Shendure, Nickerson, & Guo, 2014). Previous studies have also indicated significant genetic heterogeneity for familial TAAD as many families have not been linked to any known gene mutations leading to aneurysmal formation and they remain unidentified (Schubert, Landis, Shikany, Hinton, & Ware, 2016). As a result, clinical and genetic heterogeneity of TAA disease is challenging as a positive result for a genetic test may have a major effect on the screening of individuals who are at risk and for disease management (Schubert et al., 2016).

Potential phenotypes resulting from *SMAD3* variants can range from minimal features to those observed in Marfan syndrome, Loeyz–Dietz syndrome type III (LSD3) or even vascular Ehlers–Danlos syndrome (Loeys & Dietz, 2008; Schepers et al., 2018). In addition, pathogenic *SMAD3* variants have been found to be associated with TAA/D as well as premature osteoarthritis and skeletal abnormalities. A large number of individuals with *SMAD3* pathogenic variants do not present with an aortic event (Hostetler et al., 2019). As a result, it is generally accepted that *SMAD3* variants are characterized by inter‐ and intra‐familial variability in phenotypic expression in addition to incomplete penetrance. Moreover, some patients present vascular aneurysms or dissections of small arteries without an aortic phenotype (Chesneau et al., 2020; Hostetler et al., 2019). The absence of complete arterial tree imaging in some of our patients makes characterization of a definitive clinical phenotype rather difficult.

In this study, a novel splice site variant in *SMAD3*, c.871+1G>A, was identified and cosegregated with the affected individuals in two apparently unrelated Cypriot families with nonsyndromic familial TAA. Immediate members of the two families were asked if there was any relationship between themselves and it seems that they were not aware of any such connection. Previous studies demonstrated the association of mutations in *SMAD3* and the form of aneurysms and dissections (Hilhorst‐Hofstee et al., 2013; Van De Laar et al., 2011e Laar et al., 2011; Regalado et al., 2011) therefore, the two families were informed of the possible risk for aortic disease and a family study was initiated. Following the genetic screening, the presence of c.871+1G>A variant in *SMAD3* was also found in additional family members who have not as yet developed any clinical manifestations of the disease. Analysis of mRNA from the patient's tissue (Proband A) by RT‐PCR and Sanger sequencing confirmed aberrant splicing and the occurrence of exon 6 skipping.

In silico protein modeling using WT and MUT amino acid sequence indicated conformational changes of SMAD3 that are believed to be critical for the protein interaction and might interfere with protein function. I‐TASSER modeling shows the protein structure based on the closest similarity from the PDB (with template 1khxA). The Smad family of proteins serve as transcription factors by directly binding to specific DNA sequences and any changes in the DNA‐binding motif might affect DNA‐binding transcription factor activity. To date, 67 mutations have been identified in *SMAD3* including missense, nonsense, frameshift, and splice site mutations which are spread over the entire gene with the majority (42 out of 67) of them located in the Mad homology 2 (MH2) protein–protein binding domain (Schepers et al., 2018). Mutations located in the MH2 protein domain of SMAD3 are predicted to disrupt hydrogen‐bond formation which might affect protein–protein interactions (Milewicz et al., 2014). Also, mutations in the MH2 domain were supported to disrupt oligomerization of SMAD3 with SMAD4‐ and thus downstream SMAD‐dependent transcriptional activation (Zhang et al., 2015). In addition, MH2 domain is a region that is highly conserved among species therefore, mutations in this domain are more likely to have a deleterious effect (Van De Laar et al., 2011e Laar et al., 2011; Zhang et al., 2015). Previous studies reported that most disease‐causing variants are located within exon 6 of *SMAD3* in the MH2 domain (Van De Laar et al., 2011e Laar et al., 2011; Regalado et al., 2011; Zhang et al., 2015). The novel variant c.871+1G>A, which is a donor splice variant, leads to exon 6 skipping, resulting in an in‐frame transcript which does not lead to nonsense‐mediated decay, therefore haploinsufficiency is not indicated as the mechanism. As shown by previous studies, donor and acceptor splice site variants typically lead to a loss of function protein (Baralle & Baralle, 2005) and loss‐of‐function variants in *SMAD3* are known to be pathogenic (Aubart et al., 2014; Regalado et al., 2011). In addition, pathogenic variants that lead to protein production with alterations in the MH2 domain have shown to be more severe compared to haploinsufficiency pathogenic variants (Hostetler et al., 2019). The spanning amino acids 271–324 in exon 6 and exon 7 contribute to the proper folding of the MH2 domain and have shown to exhibit strong interaction with exportin‐4 (XPO4) that mediates SMAD3 nuclear export (Kurisaki et al., 2006). Also, the SMAD3 linker region (137–231 amino acid positions) has been previously shown to have transcriptional activity that plays an important role in the activation of several TGF‐β/Smad‐responsive reporter genes (Wang, Long, Matsuura, HE, & Liu, 2005). In addition, adjacent pathogenic variants in exon 6 and exon 7 of *SMAD3* (such as p.Arg287Trp [Van Campens et al., 2015ampens et al., 2015; De Laar et al., 2011], p.Arg287Gln [Aubart et al., 2014; Schepers et al., 2018], p.Arg288Aspfs*53 [Schepers et al., 2018], p.Arg292Aspfs*53 [Aubart et al., 2014] and p.Leu296Pro [Campens et al., 2015]) have been previously reported in families with TAAs. In addition, the c.871+2T>C variant was classified as likely pathogenic by Invitae which has been reported for TAA/D. Therefore, it can be assumed that residues in exon 6 are critical for the function of *SMAD3*. The novel c.871+1G>A variant falls within intron 6–7 in MH2 domain leading to exon 6 skipping (amino acid sequence 220–291) which also affects highly conserved amino acids (data not shown) (UniProt sequence alignment tool against the genome of five species human/chimpanzee/mouse/big/chicken).

*SMAD3* encodes a protein that is involved in cellular TGF‐β signaling pathway initiated by TGFBR1 and TGFBR2 receptors and regulates gene transcription (Regalado et al., 2011). Mutations in *SMAD3* may lead to deficiency and disruption of TGF‐β signaling pathway which were reportedly appeared to cause histological disorganization of the media layer, elastic fiber fragmentation and loss as well as collagen accumulation in the media layer which are associated with the development of aortic aneurysms (Berthet, Hanna, Giraud, & Soubrier, 2015; Chacko et al., 2004; Van De Laar et al., 2011e Laar et al., 2011; Regalado et al., 2011). *SMAD3* accounts for up to 2% of familial and nonfamilial TAA/D (Milewicz & Regalado, 2003). Pathogenic variants in *SMAD3* showed to be associated to Loeys–Dietz syndrome type III (OMIM#613795), a syndromic form of aortic aneurysms (Van De Laar et al., 2011e Laar et al., 2011). Previous studies have shown *SMAD3* gene mutations to be associated with thoracic aortic aneurysms, abdominal aortic aneurysms and to a predisposition to ICAs (Regalado et al., 2011). Also it has been supported that individuals who carry pathogenic variants in *SMAD3* are more prone to early aortic dissection and/or rupture, even in a mildly dilated aorta (Van Der Linde et al., 2012). Other clinical characteristics that have been found to be associated to *SMAD3* gene mutations include arterial tortuosity, bicuspid aortic valve (Van Der Linde et al., 2012), neurological features and immunological disorder (Aubart et al., 2014), early osteoarthritis (Van De Laar et al., 2011e Laar et al., 2011) and rheumatoid arthritis (Berthet et al., 2015). Therefore, clinical heterogeneity is observed among *SMAD3*‐familial TAA/D (Regalado et al., 2011). Based on this, the importance of molecular genetic screening to identify a causative mutation which will facilitate early diagnosis and treatment is highlighted in family cases with TAA/D.

In conclusion, in this study, we describe the molecular analysis and clinical characteristics of two Cypriot families with a novel splicing mutation in *SMAD3*. The novel variant of *SMAD3* (chr15:67473792 G>A) is a splicing mutation and multiple lines of evidence have documented the contribution of this variant for the TAA phenotype of affected individuals in the two Cypriot families. According to the ACMG guidelines the c.871+1G>A variant can be classified as pathogenic with one Very Strong (PVS1), one Strong (PS3), one Moderate (PM2) and one Supporting (PP1) criteria of pathogenicity. Our finding expands the spectrum of variants that are associated with TAA/D and further strengthens the connection between the presence of aneurysm phenotype and *SMAD3* variants. In addition, the relationship between *SMAD3* genotype and phenotype will further improve risk stratification and clinical management, identifying high‐risk subgroups. Further mutations and variants are needed to be identified for screening purposes in families with nonsyndromic familial TAA/D which may be beneficial for presymptomatic and younger family members for the prevention of sudden death of affected individuals and early intervention.

In summary, our report provides two Cypriot family cases with TAA associated with a *SMAD3* gene variant. In the two families, additional family members at risk were identified, genetic counseling was provided and appropriate cardiovascular follow‐ups with cardiologists and pediatric cardiologists was recommended.

## CONFLICT OF INTEREST

There is no conflict of interest.

## AUTHOR CONTRIBUTION

AK assisted in design of the study, carried out all the experiments and drafted the manuscript. EB assisted in the design of the study and edited the manuscript. VB diagnosed the patients and contributed to the writing of the case report. KM performed analysis of the targeted sequencing data and edited the manuscript. MS diagnosed the patients and contributed to the writing of the case report. GAT diagnosed the patients and contributed to the writing of the case report. MAC contributed to study planning and revised the final manuscript. All authors read and approved the final manuscript.

## Data Availability

The data that support the findings of this study are available on request from the corresponding authors [AK and MAC] on reasonable request. The data are not publicly available due to information that could compromise participant's privacy.

## References

[mgg31378-bib-0001] Aubart, M. , Gobert, D. , Aubart‐Cohen, F. , Detaint, D. , Hanna, N. , d’Indya, H. , … Jondeau, G. (2014). Early‐onset osteoarthritis, Charcot‐Marie‐Tooth like neuropathy, autoimmune features, multiple arterial aneurysms and dissections: An unrecognized and life threatening condition. PLoS One, 9(5), 1–8. 10.1371/journal.pone.0096387 PMC4012990 24804794

[mgg31378-bib-0002] Auwera, G. A. , Carneiro, M. O. , Hartl, C. , Poplin, R. , del Angel, G. , Levy‐Moonshine, A. , … DePristo, M. A. (2013). From FastQ data to high confidence variant calls: The Genome Analysis Toolkit best practices pipeline. Current Protocols in Bioinformatics, 11(1110), 11.10.1–11.10.33 10.1002/0471250953.bi1110s43 PMC4243306 25431634

[mgg31378-bib-0003] Baralle, D. , & Baralle, M. (2005). Splicing in action: Assessing disease causing sequence changes. Journal of Medical Genetics, 42(10), 737–749. 10.1136/jmg.2004.029538 16199547 PMC1735933

[mgg31378-bib-0004] Berthet, E. , Hanna, N. , Giraud, C. , & Soubrier, M. (2015). A case of rheumatoid arthritis associated with SMAD3 gene mutation: A new clinical entity? Journal of Rheumatology, 42(3), 556 10.3899/jrheum.140645 25729048

[mgg31378-bib-0005] Brownstein, A. J. , Kostiuk, V. , Ziganshin, B. A. , Zafar, M. A. , Kuivaniemi, H. , Body, S. C. , Elefteriades, J. A. (2018). Genes associated with thoracic aortic aneurysm and dissection: 2018 update and clinical implications. Aorta, 6(1), 13–20. 10.1055/s-0038-1639612 30079932 PMC6136681

[mgg31378-bib-0006] Brownstein, A. J. , Ziganshin, B. A. , Kuivaniemi, H. , Simon, C. , Bale, A. E. , & Elefteriades, J. A. (2017). Genes associated with thoracic aortic aneurysm and dissection: An update and clinical implications. Aorta, 5(1), 11–20. 10.12945/j.aorta.2017.17.003 28868310 PMC5570562

[mgg31378-bib-0007] Campens, L. , Callewaert, B. , Muiño Mosquera, L. , Renard, M. , Symoens, S. , De Paepe, A. , … De Backer, J. (2015). Gene panel sequencing in heritable thoracic aortic disorders and related entities ‐ Results of comprehensive testing in a cohort of 264 patients. Orphanet Journal of Rare Diseases, 10(9), 1–9. 10.1186/s13023-014-0221-6 25644172 PMC4326194

[mgg31378-bib-0008] Chacko, B. M. , Qin, B. Y. , Tiwari, A. , Shi, G. , Lam, S. , Hayward, L. J. , Lin, K. (2004). Structural basis of heteromeric smad protein assembly in TGF‐b signaling Benoy. Molecular Cell, 15, 813–823. 10.1016/j.molcel.2004.07.016 15350224

[mgg31378-bib-0009] Chesneau, B. , Edouard, T. , Dulac, Y. , Colineaux, H. , Langeois, M. , Hanna, N. , … Plaisancié, J. (2020). Clinical and genetic data of 22 new patients with SMAD3 pathogenic variants and review of the literature. Molecular Genetics and Genomic Medicine, 8(5), 1–9. 10.1002/mgg3.1132 PMC7216810 32154675

[mgg31378-bib-0010] Desmet, F. O. , Hamroun, D. , Lalande, M. , Collod‐Bëroud, G. , Claustres, M. , & Béroud, C. (2009). Human Splicing Finder: An online bioinformatics tool to predict splicing signals. Nucleic Acids Research, 37(9), 1–14. 10.1093/nar/gkp215 19339519 PMC2685110

[mgg31378-bib-0011] Gagnier, J. J. , Kienle, G. , Altman, D. G. , Moher, D. , Sox, H. , Riley, D. , … Tugwell, P. (2013). The CARE guidelines: Consensus‐based clinical case reporting guideline development. Global Advances in Health and Medicine, 2(5), 38–43. 10.7453/gahmj.2013.008 PMC3833570 24416692

[mgg31378-bib-0012] Hannuksela, M. , Stattin, E. , Klar, J. , Ameur, A. , Johansson, B. , Sörensen, K. , & Carlberg, B. (2016). A novel variant in MYLK causes thoracic aortic dissections: Genotypic and phenotypic description. BMC Medical Genetics, 17(1), 1–9. 10.1186/s12881-016-0326-y 27586135 PMC5008005

[mgg31378-bib-0013] Hebsgaard, S. M. , Korning, P. G. , Tolstrup, N. , Engelbrecht, J. , Rouzé, P. , & Brunak, S. (1996). Splice site prediction in Arabidopsis thaliana pre‐mRNA by combining local and global sequence information. Nucleic Acids Research, 24(17), 3439–3452. 8811101 10.1093/nar/24.17.3439 PMC146109

[mgg31378-bib-0014] Hilhorst‐Hofstee, Y. , Scholte, A. J. H. A. , Rijlaarsdam, M. E. B. , van Haeringen, A. , Kroft, L. J. , Reijnierse, M. , … Breuning, M. H. (2013). An unanticipated copy number variant of chromosome 15 disrupting SMAD3 reveals a three‐generation family at serious risk for aortic dissection. Clinical Genetics, 83(4), 337–344. 10.1111/j.1399-0004.2012.01931.x 22803640

[mgg31378-bib-0015] Hostetler, E. M. , Regalado, E. S. , Guo, D. , Hanna, N. , Arnaud, P. , Muiño‐mosquera, L. , Milewicz, D. M. (2019). SMAD3 pathogenic variants: Risk for thoracic aortic disease and associated complications from the Montalcino Aortic Consortium. 1–9. 10.1136/jmedgenet-2018-105583 30661052

[mgg31378-bib-0016] Jondeau, G. , & Boileau, C. (2014). Familial thoracic aortic aneurysms. Current Opinion in Cardiology, 29(6), 492–498. 10.1097/HCO.0000000000000114 25290696

[mgg31378-bib-0017] Karczewski, K. J. , Francioli, L. C. , Tiao, G. , Cummings, B. B. , Alföldi, J. , Wang, Q. , … MacArthur, D. G. (2019). Variation across 141,456 human exomes and genomes reveals the spectrum of loss‐offunction intolerance across human protein‐coding genes. BioRxiv, 531210, 10.1101/531210

[mgg31378-bib-0018] Keravnou, A. , Bashiardes, E. , Michailidou, K. , Soteriou, M. , Moushi, A. , & Cariolou, M. (2018). Novel variants in the ACTA2 and MYH11 genes in a Cypriot family with thoracic aortic aneurysms: A case report. BMC Medical Genetics, 19, 1–8. 10.1186/s12881-018-0728-0 PMC6286578 30526509

[mgg31378-bib-0019] Kurisaki, A. , Kurisaki, K. , Kowanetz, M. , Sugino, H. , Yoneda, Y. , Heldin, C.‐H. , & Moustakas, A. (2006). The mechanism of nuclear export of Smad3 involves exportin 4 and Ran. Molecular and Cellular Biology, 26(4), 1318–1332. 10.1128/MCB.26.4.1318-1332.2006 16449645 PMC1367208

[mgg31378-bib-0020] Landenhed, M. , Engström, G. , Gottsäter, A. , Caulfield, M. P. , Hedblad, B. O. , Newton‐Cheh, C. , … Smith, J. G. (2015). Risk profiles for aortic dissection and ruptured or surgically treated aneurysms: A prospective cohort study. Journal of the American Heart Association, 4(1), e001513 10.1161/JAHA.114.001513 25609416 PMC4330075

[mgg31378-bib-0021] Lempel, J. K. , Frazier, A. A. , Jeudy, J. , Kligerman, S. J. , Schultz, R. , Ninalowo, H. A. , … White, C. S. (2014). Aortic arch dissection: A controversy of classification. Radiology, 271(3), 848–855. 10.1148/radiol.14131457 24617732

[mgg31378-bib-0022] Li, H. (2011). A statistical framework for SNP calling, mutation discovery, association mapping and population genetical parameter estimation from sequencing data. Bioinformatics, 27(21), 2987–2993. 10.1093/bioinformatics/btr509 21903627 PMC3198575

[mgg31378-bib-0023] Li, H. , & Durbin, R. (2009). Fast and accurate short read alignment with Burrows‐Wheeler transform. Bioinformatics (Oxford, England), 25(14), 1754–1760. 10.1093/bioinformatics/btp324 PMC2705234 19451168

[mgg31378-bib-0024] Loeys, B. L. , & Dietz, H. C. (2008).Loeys‐Dietz Syndrome. GeneReviews® [Internet]. Retrieved from https://www.ncbi.nlm.nih.gov/books/NBK1133/

[mgg31378-bib-0025] Milewicz, D. M. , & Regalado, E. (2003).Heritable Thoracic Aortic Disease Overview. GeneReviews® [Internet]. Retrieved from https://www.ncbi.nlm.nih.gov/books/NBK1120/

[mgg31378-bib-0026] Milewicz, D. M. , Regalado, E. , Shendure, J. , Nickerson, D. A. , & Guo, D. (2014). Successes and challenges of using whole exome sequencing to identify novel genes underlying an inherited predisposition for thoracic aortic aneurysms and acute aortic dissections. Trends in Cardiovascular Medecine, 24(2), 53–60. 10.1016/j.tcm.2013.06.004 PMC3917689 23953976

[mgg31378-bib-0027] Reese, M. G. , Eeckman, F. H. , Kulp, D. , & Haussler, D. (1997). Improved Splice Detection in Genie. Journal of Computational Biology, 4(3), 311–323. 9278062 10.1089/cmb.1997.4.311

[mgg31378-bib-0028] Regalado, E. S. , Guo, D. , Villamizar, C. , Avidan, N. , Gilchrist, D. , Mcgillivray, B. , … Bertoli‐Avella, A. M. (2011). Exome sequencing identifies SMAD3 mutations as a cause of familial thoracic aortic aneurysm and dissection with intracranial and other arterial aneurysms. Circulation Research, 109(6), 680–686. 10.1161/CIRCRESAHA.111.248161 21778426 PMC4115811

[mgg31378-bib-0030] Richards, S. , Aziz, N. , Bale, S. , Bick, D. , Das, S. , Gastier‐Foster, J. , … Rehm, H. L. (2015). Standards and guidelines for the interpretation of sequence variants: A joint consensus recommendation of the American College of Medical Genetics and Genomics and the Association for Molecular Pathology. Genetics in Medicine, 17(5), 405–424. 10.1038/gim.2015.30 25741868 PMC4544753

[mgg31378-bib-0031] Schepers, D. , Tortora, G. , Morisaki, H. , MacCarrick, G. , Lindsay, M. , Liang, D. , … Loeys, B. (2018). A mutation update on the LDS‐associated genes TGFB2/3 and SMAD2/3. Human Mutation, 39(5), 621–634. 10.1002/humu.23407 29392890 PMC5947146

[mgg31378-bib-0032] Schubert, J. A. , Landis, B. J. , Shikany, A. R. , Hinton, R. B. , & Ware, S. M. (2016). Clinically Relevant Variants Identified in Thoracic Aortic Aneurysm Patients by research exome sequencing. American Journal of Medical Genetics, Part A, 170A(5), 1288–1294. 10.1002/ajmg.a.37568 26854089 PMC5125072

[mgg31378-bib-0033] van de Laar, I. M. B. H. , Oldenburg, R. A. , Pals, G. , Roos‐Hesselink, J. W. , de Graaf, B. M. , Verhagen, J. M. A. , … Bertoli‐Avella, A. M. (2011). Mutations in SMAD3 cause a syndromic form of aortic aneurysms and dissections with early‐onset osteoarthritis. Nature Genetics, 43(2), 121–126. 10.1038/ng.744 21217753

[mgg31378-bib-0034] van der Linde, D. , van de Laar, I. M. B. H. , Bertoli‐Avella, A. M. , Oldenburg, R. A. , Bekkers, J. A. , Mattace‐Raso, F. U. S. , … Roos‐Hesselink, J. W. (2012). Aggressive cardiovascular phenotype of aneurysms‐osteoarthritis syndrome caused by pathogenic SMAD3 variants. Journal of the American College of Cardiology, 60(5), 397–403. 10.1016/j.jacc.2011.12.052 22633655

[mgg31378-bib-0035] Verstraeten, A. , Luyckx, I. , & Loeys, B. (2017). Aetiology and management of hereditary aortopathy. Nature Reviews Cardiology, 14, 197–208. 10.1038/nrcardio.2016.211 28102232

[mgg31378-bib-0036] Wang, G. , Long, J. , Matsuura, I. , He, D. , & Liu, F. (2005). The Smad3 linker region contains a transcriptional activation domain. Biochemical Journal, 386(1), 29–34. 10.1042/bj20041820 15588252 PMC1134763

[mgg31378-bib-0037] Yang, J. , & Zhang, Y. (2015). I‐TASSER server: New development for protein structure and function predictions. Nucleic Acids Research, 43(W1), W174–W181. 10.1093/nar/gkv342 25883148 PMC4489253

[mgg31378-bib-0038] Zhang, C. , Freddolino, P. L. , & Zhang, Y. (2017). COFACTOR: Improved protein function prediction by combining structure, sequence and protein‐protein interaction information. Nucleic Acids Research, 45(W1), W291–W299. 10.1093/nar/gkx366 28472402 PMC5793808

[mgg31378-bib-0039] Zhang, W. , Zhou, M. , Liu, C. , Leu, C. , Qiao, T. , Huang, D. , … Liu, Z. (2015). A novel mutation of SMAD3 identified in a Chinese family with aneurysms‐osteoarthritis syndrome. BioMed Research International, 2015(968135), 1–6. 10.1155/2015/968135 PMC4499615 26221609

